# The Antiacetylcholinesterase and Antileishmanial Activities of *Canarium patentinervium* Miq.

**DOI:** 10.1155/2014/903529

**Published:** 2014-05-15

**Authors:** R. Mogana, A. Adhikari, S. Debnath, S. Hazra, B. Hazra, K. Teng-Jin, C. Wiart

**Affiliations:** ^1^School of Pharmacy, Faculty of Science, Center for Natural and Medicinal Products Research, University of Nottingham (Malaysia Campus), Jalan Broga, 43500 Semenyih, Selangor, Malaysia; ^2^HEJ Research Institute of Chemistry, University of Karachi, Karachi 75270, Pakistan; ^3^Department of Pharmaceutical Technology, Jadavpur University Calcutta, West Bengal 700032, India

## Abstract

In continuation of our natural and medicinal research programme on tropical rainforest plants, a bioassay guided fractionation of ethanolic extract of leaves of *Canarium patentinervium* Miq. (Burseraceae Kunth.) led to the isolation of scopoletin **(1)**, scoparone **(2)**, (+)-catechin **(3)**, vomifoliol **(4)**, lioxin **(5)**, and syringic acid **(6)**. All the compounds exhibited antiacetylcholinesterase activity with syringic acid, a phenolic acid exhibiting good AChE inhibition (IC_50_ 29.53 ± 0.19 **μ**g/mL). All compounds displayed moderate antileishmanial activity with scopoletin having the highest antileishmanial activity (IC_50_ 163.30 ± 0.32 **μ**g/mL). Given the aforementioned evidence, it is tempting to speculate that *Canarium patentinervium* Miq. represents an exciting scaffold from which to develop leads for treatment of neurodegenerative and parasitic diseases.

## 1. Introduction

Neurodegenerative disease is a term applied to a variety of conditions arising from a chronic breakdown and deterioration of the neurons, particularly those of the central nervous system. Alzheimer's disease (AD) was first described in 1906 by a Bavarian neuropsychiatrist Alois Alzheimer [1]. It is a complex, multifactorial, progressive, neurodegenerative disease primarily affecting the elderly population and is estimated to account for 50–60% of dementia cases in persons over 65 years of age [2]. The pathophysiology of AD is complex and involves several different biochemical pathways. The first neurotransmitter defect discovered in AD involved acetylcholine (ACh), which plays an important role in memory and learning. However, in patients with AD, the ACh which is released has a very short half-life due to the presence of large amounts of the enzymes: acetyl cholinesterase (AChE) and butyrylcholinesterase (BChE), which are both present in the brain and are detected among neurofibrillary tangles and neuritic plaques [3]. These enzymes hydrolyse the ester bond in the ACh molecule, leading to loss of stimulatory activity. Hodges [4] demonstrated that the inhibition of AChE holds a key role not only to enhance cholinergic transmission in the brain but also to reduce the aggregation of *β*-amyloid and the formation of the neurotoxic fibrils in AD. Therefore, AChE and BChE inhibitors have become remarkable alternatives in treatment of AD [5]. Existing anticholinesterase drugs (for example, tacrine, donepezil, physostigmine, galanthamine, and heptylphysostigmine) for the treatment of dementia are reported to have several dangerous adverse effects such as hepatotoxicity, short duration of biological action, low bioavailability, adverse cholinergic side effects in the periphery, and a narrow therapeutic window [6]. This necessitates the interest in finding better AChE inhibitors from natural resources.

The neurotransmitter ACh is synthesised in presynaptic cholinergic neurons by choline acetyltransferase (CAT or ChAT). The process entails transfer of an acetyl group from acetyl-coenzyme A to choline. The choline released in the process is reused in synthesizing new ACh. Inhibition of AChE increases the amount of ACh available for neurotransmission. Choline is the precursor of phosphatidylcholine (PC), a main component of* Leishmania* promastigote membranes [7]. Therefore, inhibition of choline formation may decrease* Leishmania* survival. This hypothesis can be tested by using inhibitors of the acetylcholinesterase enzyme (AChE), which catalyzes the hydrolysis of acetylcholine to choline and acetic acid, as leishmanicidal compounds. This may identify another mechanism of action for antileishmanial activity [8].

Leishmaniasis is still one of the most neglected diseases in the world. During the last 10 years, many scientific studies involving this disease have been related to treatment strategies and led to a reduction in drug prices; however, the morbidity and mortality of this disease has continued to increase worldwide [9]. For more than 50 years, the traditional chemotherapy used to treat leishmaniasis has been based on the use of pentavalent antimonial drugs. However, the toxicity of these agents and their side effects, along with the development of resistance and differences in strain sensitivity, are challenges that must be overcome [10].

*Canarium patentinervium* Miq. is a rare plant from the family Burseraceae and genus* Canarium* found in the Asia Pacific region previously recorded for its usage in wound healing by the indigenous people of Malaysia. In continuation to our earlier studies on the pharmacological properties of* Canarium patentinervium* Miq. [11, 12], this study investigates the antiacetylcholinesterase and antileishmanial activities of the plant. To the best of our knowledge, this is the first comprehensive study on isolated compounds from* Canarium patentinervium* Miq. investigating the antiacetylcholinesterase and antileishmanial activities.

## 2. Materials and Method

### 2.1. Plant Material

The leaves and barks of* Canarium patentinervium* Miq. were collected from one individual tree from Bukit Putih, Selangor, Malaysia (3°5′24′′N 101°46′0′′E). The plant was identified by Mr. Kamaruddin (Forest Research Institute of Malaysia). A herbarium sample (PID 251210-12) has been deposited in the Forest Research Institute of Malaysia. The leaves were air dried and grinded into small particles using an industrial grinder.

### 2.2. Chemicals and Reagents

5,5′-Dithio-bis(2-nitrobenzoic) acid (DTNB), galanthamine, electric eel acetylcholinesterase (Ache) (Type-VI-S, EC 3.1.1.7), and amphotericin B were purchased from Sigma Aldrich, St. Louis, MO, USA. Hexane, ethyl acetate, dichloromethane, chloroform (analytical grade), ammonium bicarbonate, acetonitrile, and ethyl acetate (HPLC grade) were purchased from Friendemann Schmidt Chemicals. Methanol, ethanol 95%, and DMSO were from R&M Marketing, Essex, UK. Acetylthiocholine iodide was purchased from Calbiochem; silica gel and preparative thin layer chromatography plates (0.5 and 2 mm thickness) were purchased from Merck. Sephadex LH-20 was purchased from GE Healthcare. MTT [3-(4,5-dimethylthiazol-2-yl)-2,5-diphenyl tetrazolium bromide] was purchased from Sisco Research Laboratory, Mumbai, India.

### 2.3. Extraction and Isolation

**Figure 1:**
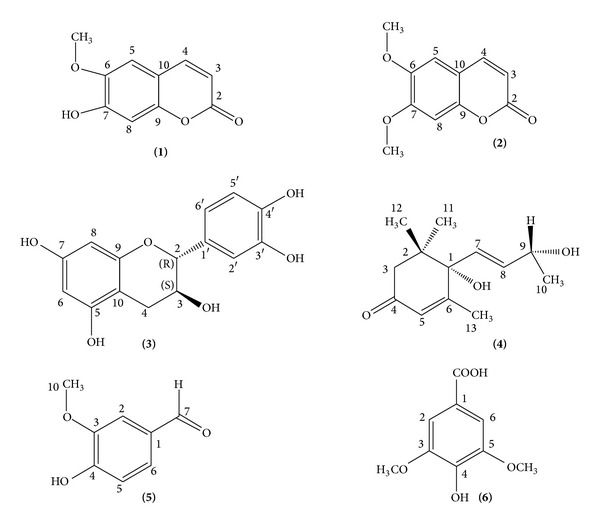
Chemical structures of isolated compounds from* Canarium patentinervium* Miq.

Dried and grinded sample of leaves (2.8 kg) was soaked in hexane with the ratio of 1 : 3 parts of sample to solvent for 2 h in a 60°C water bath then filtered and concentrated with a rotary evaporator (Buchi, R-200 Switzerland). This was repeated 3 times. Thereafter the leaves and barks were left to air dry completely for 3 days before repeating the whole process with chloroform and then ethanol, respectively. The yield for the hexane, chloroform, and ethanol extract of leaves was 1.25%, 1.11%, and 6.45%, respectively. The ethanol extract of the leaves (80 g) was then partitioned with petroleum ether, chloroform, and water to yield the respective solvent extracts. The chloroform extract (5 g) was further purified by silica gel chromatography (4 cm × 90 cm, 0.063–0.200 mesh) and eluted with a chloroform/methanol gradient elution (the ratio from 100 : 0 to 8 : 100). Thirteen column fractions were collected and analysed by TLC (chloroform/methanol). Fractions with similar TLC pattern were combined to total of four fractions. Fraction B that was yielded from chloroform/methanol ratio 100 : 4 was rechromatographed on a preparative TLC (2 mm thickness) with solvent system chloroform/methanol (ratio of 1000 : 15) yielding total 7 bands. Band three was collected and rechromatographed on preparative TLC (0.5 mm thickness) with solvent system chloroform/methanol (ratio of 89 : 11) to yield four bands, with band two yielding compound** 1** (49 mg) and band three yielding compound** 2** (11 mg) (Figure 1). The water extract (24 g) was further purified by Sephadex LH-20 with mobile phase ethanol yielding 16 fractions which was then recombined to four fractions. Fraction B was loaded on Sephadex LH-20 with mobile phase methanol yielding 3 fractions, whereby fraction B was compound** 3** (14 mg). Crude chloroform extract (7.2 g) was dissolved in dichloromethane : methanol (2 : 1) and subjected to PTLC with mobile phase ethyl acetate : methanol (10 : 1) yielding 3 bands. Bands 1 and 2 were run on semipreparative HPLC with mobile phase ethyl acetate : acetonitrile (6 : 1) which yielded compound** 4** (15 mg) at retention time (Rt) = 14.1 min and compound** 5** (3.4 mg) at Rt = 15.8. Band 3 was partitioned between water and ethyl acetate and the aqueous layer was run on semipreparative HPLC with mobile phase ammonium bicarbonate : acetonitrile. Compound** 6** (3.0 mg) was eluted at Rt = 3.3 min.

### 2.4. Antiacetylcholinesterase Assay

Acetylcholinesterase (AChE) inhibitory activity was measured by slightly modifying the spectrophotometric method developed by Ellman et al. [13]. 5,5′-Dithio-bis(2-nitrobenzoic) acid (DTNB) was used for the measurement of anti-AChE activity. All the other reagents and conditions were the same as described previously [10]. Test sample and galanthamine which were used as positive control were dissolved in dimethyl sulfoxide (DMSO, R and M) prior to assay at a stock concentration of 5 mM, and serial dilution was done accordingly to obtain a good EC_50_ curve. In brief, 130 *μ*L of 0.1 mM sodium phosphate buffer (pH 8.0), 20 *μ*L of DTNB, 20 *μ*L of test solution, and 20 *μ*L of AChE solution were added by multichannel automatic pipette (Eppendorf, Germany) in a 96-well microplate and incubated for 15 min at 25°C. The reaction was then initiated with the addition of 10 *μ*L of acetylthiocholine iodide. The hydrolysis of acetylthiocholine iodide was monitored by the formation of the yellow 5-thio-2-nitrobenzoate anion as a result of the reaction of DTNB with thiocholines, catalysed by enzymes at a wavelength of 412 nm utilizing a 96-well microplate Thermo Scientific Varioskan Flash microtiter plate reader, and linked to a computer equipped with SkanIt Software 2.4.3. Percentage inhibition of AChE was determined by comparison of rates of reaction of samples relative to blank sample (ethanol in phosphate buffer pH = 8) using the formula (*E* − *S*)/*E* × 100, where *E* is the activity of enzyme without test sample and *S* is the activity of enzyme with test sample. The experiments were done in triplicate. Galanthamine was used as reference.

### 2.5. Antileishmanial Assay

The study on the antileshmanial activity of the extracts and isolated compounds was performed according to the standard methods as described by Mosmann [14] against* Leishmania donovani* (strain MHOM/IN/1983/AG83) promastigotes by using MTT colorimetric assay. Amphotericin B was used as the positive control in all the experiments. Promastigotes (5 × 10^5^ cells/mL; 300 *μ*L) were treated with and without tested samples at concentrations of 100 and 500 *μ*g/mL and incubated at 22 ± 2°C. After 72 hr, cells were harvested and resuspended in PBS (500 *μ*L) containing MTT (0.3 mg/mL). Purple formazan crystals were dissolved in DMSO and the optical density (O.D.) was measured at 570 nm in an ELISA reader (BIO-RAD; model 680, USA). The number of viable cells was directly proportional to the amount of formazan produced through the reduction of yellow MTT by the dehydrogenase enzymes present in the inner mitochondrial membrane of the living cells. The percentage of growth inhibition was calculated as follows: %inhibition = [(O.D. of untreated control − O.D. of treated set)/O.D. of untreated control] × 100.

### 2.6. Statistical Analysis

Concentration-response curves were calculated using the Prism software package 5.00 for Windows, GraphPad Software, San Diego, CA, USA, http://www.graphpad.com/ (GraphPad, San Diego, USA) and data were reported as mean and SD values obtained from a minimum of three determinations. Nonlinear best fit was plotted with SD and 95% confidence interval. All data were expressed as mean ± standard deviation. Data were analysed using one-way Anova followed by Tukey test using GraphPad Prism5 software. A significant difference was considered at the level of *P* < 0.01.

## 3. Results and Discussions

### 3.1. Isolated Compounds

Six compounds were isolated for the first time from* Canarium patentinervium* Miq. using various isolation techniques such as TLC, CC, and HPLC and identified with NMR method and in comparison to the literature. Compounds isolated scopoletin, scoparone, (+)-catechin, lioxin, and syringic acid were phenolics while vomifoliol was a norsesquiterpene with a cyclohexenone ring. Lioxin, syringic acid, and vomifoliol were isolated from this genus* Canarium* for the first time (Figure 1). Catechin exists in nature as (+) and (−) enantiomers. The optical rotation of the compound in methanol was tested using Schmidt + Haensch Polartronic H532 Polarimeter. The experimental rotation was an average of +69.25 (*n* = 4) concluding that this compound is (+)-catechin. Previous published data on (+)-catechin reports an optical rotation of +56.60 [15].

Scopoletin [16]: pale yellow powder; ^1^H-NMR (400 MHz, CDCl_3_) *δ*; 3.98 (6-OCH3, s, 3H), 6.30 (H-3, d, *J* = 9.5 Hz, 1H), 6.87 (H-5, s, 1H), 6.95 (H-8, s, 1H), 7.63 (H-4, d, *J* = 9.5 Hz, 1H); ^13^C-NMR (125 MHz, CDCl_3_) *δ*; 56.4 (6-OCH3), 103.2 (C-5), 107.4 (C-8), 111.6 (C-3), 113.5 (C-10), 143.3 (C-4), 144.0 (C-6), 149.7 (C-9), 150.2 (C-7), 161.6 (C-2); ESI-MS:* m/z* (relative intensity, %): 192 (M^+^, 100), 177 (70), 164 (28) 149 (59).

Scoparone [17]: pale yellow powder; ^1^H-NMR (500 MHz, CDCl_3_) *δ*; 3.98 (7-OCH3, s, 3H), 3.95 (6-OMe, s, 3H), 6.32 (H-3, d, *J* = 9.6 Hz, 1H), 6.88 (H-8, s, 1H), 6.87 (H-5, s, 1H), 7.64 (H-4, d, *J* = 9.6 Hz, 1H); ^13^C-NMR (125 MHz, CD3Cl) *δ*; 56.00 (6-OCH3), 56.40 (7-OCH3), 100.05 (C-5), 107.98 (C-10), 111.45 (C-3), 113.59 (C-9), 143.28 (C-8), 146.37 (C-7), 150.06 (C-4), 152.87 (C-6), 161.41 (C-2); ESI-MS:* m/z* (relative intensity, %): 206 (M^+^100), 191 (39.7), 178 (17.9), 163 (28.9), 149 (6.7), 135 (17.2), 107 (12.4), 79 (11.3).

(+)-Catechin [18]: slightly pale yellow needles; ^1^H-NMR (500 MHz, CD_3_OD) *δ*; 2.52 (H-10, dd, 1H), 2.52, 2.87 (H-4, dd, 1H), 3.98 (H-3, m, 1H), 4.01 (H-2, d, 1H), 4.58 (7-OH, d, 1H), 5.87 (H-8, d, 1H), 5.94 (H-6, d, 1H), 6.74 (H-6′, dd, 1H), 6.78 (H-5′, d, 1H), 6.85 (H-2′, d, 1H); ^13^C-NMR (125 MHz, CD3OD) *δ*; 27.12 (C-4), 67.41 (C-3), 81.46 (C-2), 94.08 (C-8), 94.86 (C-6), 99.40 (C-10), 113.84 (C-2′), 114.66 (C-5′), 130.82 (C-1′), 144.83 (C-3′), 144.85 (C-4′), 155.52 (C-9), 156.19 (C-5), 156.45 (C-7); ESI-MS:* m/z* (relative intensity, %): 290 (M^+^100), 291 (17), 292 (1.3).

Vomifoliol [19, 20]: white solid; ^1^H-NMR (CD_3_OD, 500 MHz) *δ*; 1.03 (H-11, s, 3H), 1.06 (H-12, s, 3H), 1.26 (H-10, d, 3H), 1.94 (H-13, s, 3H), 2.18 (3a, d, 1H), 2.54 (3b, d, 1H), 4.34 (H-9, m, 1H), 5.81 (H-5, m, 1H), 5.82 (H-8, m, 1H), 5.90 (H-5, m, 1H); ^13^C-NMR (125 MHz, CD_3_OD) *δ*; 18.15 (C-13), 22.05 (C-12), 22.41 (C-10), 23.06 (C-11), 41.03 (C-2), 67.34 (C-9), 78.34 (C-1), 125.69 (C-5), 128.71 (C-7), 135.49 (C-8), 166.10 (C-6), 199.85 (C-4); ESI-MS:* m/z* (relative intensity, %): 224 (M^+^100), 225 (14.2), 225 (1.8).

Lioxin [21]: slightly pale yellow needles; ^1^H-NMR (500 MHz, CD_3_OD) *δ*; 3.80 (10-OCH3, s, 3H), 6.77 (OH, s, 1H), 7.30 (H-5, d, 1H), 7.30, 7.31 (H-2, H-6, m, 2H), 9.57 (H-7, s, 1H); ^13^C-NMR (125 MHz, CD_3_OD) *δ*; 54.8 (10-OCH3), 110.07 (C-2), 115.51 (C-5), 125.64 (C-6), 127.72 (C-1), 149.09 (C-3), 147.08 (C-4), 191.60 (C-7); ESI-MS:* m/z* (relative intensity, %): 152 (M^+^100), 137 (6), 123 (13).

Syringic acid [22, 23]: pale yellow powder; ^1^H NMR (500 MHz, D_2_O): 7.11 (H-2, H-6, s, 2H), 3.37 (2-OCH3, 6-OCH3, s, 3H); ^13^C NMR (125 MHz, D_2_O): 56.26 (6-OCH3), 106.92 (C-2, C-6), 121.08 (C-1), 147.08 (C-3, C-5), 148.41 (C-4), 174.79 (COOH); EI-MS:* m/z* (relative intensity, %) 198 (M+, 100), 183 (36), 168 (15), 153 (12), 127 (18), 97 (30), 83 (36), 71 (38), 69 (43).

### 3.2. The Antiacetylcholinesterase Activity

**Figure 2:**
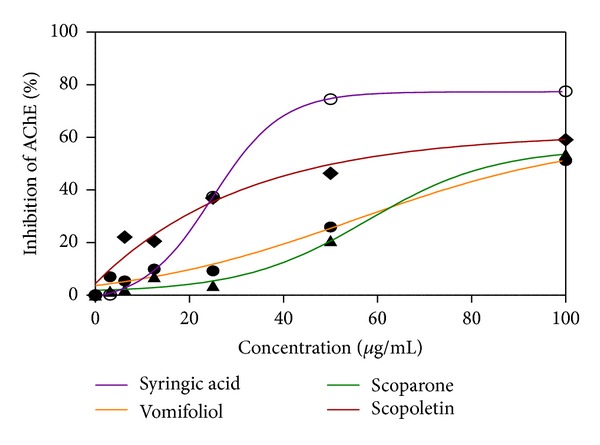
AChE inhibition by isolated compounds from* Canarium patentinervium* Miq.

In the antiacetylcholinesterase assay, chloroform extract of the barks displayed the best activity (IC_50_ = 88.59 ± 0.14 *μ*g/mL) as opposed to galanthamine (IC_50_ = 0.74 ± 0.06 *μ*g/mL). The ethanol extract of barks and leaves follows through with IC_50_ = 186.00 ± 0.15 *μ*g/mL and IC_50_ = 201.24 ± 0.15 *μ*g/mL, resp. Hexane extracts of bark and leaves and the chloroform extract of leaves had the lowest enzyme inhibition activity (IC_50_ = 570.00 ± 0.08 *μ*g/mL, IC_50_ = 842.00 ± 0.25 *μ*g/mL, and IC_50_ = 1780.00 ± 0.24 *μ*g/mL, resp.). Thus, the potency of activity against AChE was chloroform extract of barks > ethanol extract of barks > ethanol extract of leaves > hexane extract of barks > hexane extract of leaves > chloroform extract of leaves (Table 1). The antiacetylcholinesterase values for isolated compounds are shown in Table 1 and Figure 2. Only 4 compounds showed moderate enzyme inhibition, namely, syringic acid (IC_50_ =  29.53 ± 0.19 *μ*g/mL), scopoletin (IC_50_ = 51.00 ± 0.02 *μ*g/mL), scoparone (IC_50_ = 86.58 ± 0.05 *μ*g/mL), and vomifoliol (IC_50_ = 96.64 ± 0.09 *μ*g/mL) as shown in Figure 2. Flavonoid (+)-catechin showed poor enzyme inhibition with IC_50_ values > 100 *μ*g/mL.

This assay measures the inhibition activity against AChE, which is the key enzyme in the hydrolysis of acetylcholine that is responsible for muscle and organ relaxations. Acetyl cholinesterase inhibitors are therefore used medicinally to treat myasthenia gravis to increase neuromuscular transmission and to treat Alzheimer's disease (deficiency in the production of acetylcholine). Furthermore, oxidative and inflammatory processes are among the pathological features associated with the central nervous system in Alzheimer's disease [24]. The brain of patients suffering from AD is said to be under oxidative stress as a result of perturbed ionic calcium balances within their neurons and mitochondria [25]. Accumulating evidence suggests that oxidative damage to neurons plays an important role in the AD pathogenesis [26]. Because of the unclear pathogenesis of AD, there have been several hypothesis associated with the disease such as amyloid-*β* peptide-containing plaque formation, excess metal ions, oxidative stress, and reduced acetylcholine levels.

Thus, efforts to reduce oxidative injury may prove beneficial in retarding or preventing the onset and progression of AD in patients. In previous studies [11], chloroform extract of barks had displayed good antioxidant potential via nonenzymatic assays. In addition, the anti-acetylcholinesterase activity exhibited in this study (IC_50_ = 88.59 ± 0.14 *μ*g/mL) suggest that the chloroform extract of the barks hold lead compounds that inhibit acetylcholinesterase activity as well as reduce the oxidative stress with possible neuroprotective effects. This was somewhat evident with the isolation of syringic acid that had the lowest AChE inhibition among the isolated compounds (IC_50_ = 29.53 ± 0.19 *μ*g/mL). Up to date, quite a lot of studies have reported affirmative effects of phenolics in neurodegenerative diseases depending upon their antioxidative properties [27]. However, there has been a small number of data on AChE inhibitory activities of phenolic compounds. Among the phenolics isolated in this study only syringic acid, a phenolic acid, exhibited good AChE inhibition (IC_50_ = 29.53 ± 0.19 *μ*g/mL), lowest of all compounds tested. The literature concerning the role of phenolic acids and their derivatives in the neuroprotection of the CNS is, however, still incomplete. Thus the actual mechanism of inhibition needs to be investigated.

Only scopoletin, scoparone, vomifoliol, and syringic acid showed AChE inhibition at IC_50_ < 100 *μ*g/mL. Studies have also shown that naturally occurring as well as the chemically synthesised coumarin analogs exhibit potent AChE inhibitory activity [28]. Coumarin ring seems to be essential for the optimal activity and its replacement with related structural moiety such as chromone is associated with loss of AChE inhibitory activity. The substituents at coumarin moiety particularly at 6th and 7th positions also influence the activity in a significant manner. The presence of electron-donating groups such as –OCH_3_, –OH, and –NH_2_ increase the activity and it has been generally attributed to an increase in lipophilicity of compounds [29]. This might explain good activity of scopoletin (IC_50_ = 51.00 ± 0.02 *μ*g/mL) and scoparone (IC_50_ = 86.58 ± 0.05 *μ*g/mL) as both have electron donating groups at 6th and 7th positions. The presence of bulkier substituents at 6th and 7th positions of the coumarin is associated with significant loss in AChE inhibitory activity indicating the critical role of electronic as well as steric effects in influencing the AChE inhibitory activity [29]; this explains why scoparone has higher IC_50_ due to it having two bulkier methoxy groups. Cyclohexenone derivatives, vomifoliol had moderate AChE inhibition (IC_50_ = 96.64 ± 0.09 *μ*g/mL), which is to the best of our knowledge the first to be reported.

### 3.3. The Antileishmanial Activity

The result of the* in vitro* effect of extracts on* Leishmania donovani* is summarized in Table 2. The potency of extracts against* Leishmania donovani* was hexane extract of leaves > hexane extract of barks > chloroform extract of barks > chloroform extract of leaves. Both ethanol extracts of leaves and barks had IC_50_ values of above 500 *μ*g/mL. Hexane extract displayed lowest IC_50_ value of 257.40 ± 0.30 *μ*g/mL. Compound scopoletin was more potent against* Leishmania donovani* (IC_50_ = 163.30 ± 0.32 *μ*g/mL). Sensitivity of* Leishmania donovani* is then followed by the presence of lioxin (IC_50_ = 211.48 ± 0.32 *μ*g/mL), vomifoliol (IC_50_ = 302.80 ± 0.33 *μ*g/mL), scoparone (IC_50_ = 329.90 ± 0.32 *μ*g/mL), and (+)-catechin (IC_50_ = 478.93 ± 0.28 *μ*g/mL). Syringic acid was not tested for antileishmanial activity due to low yield. In the present study, six different extracts of* Canarium patentinervium* Miq. (Burseraceae) leaves and barks were screened for their* in vitro* antiparasitic activities; among the different extracts tested, the hexane extract of leaves showed moderate antileishmanial activity with IC_50_ values of 257.40 ± 0.30 *μ*g/mL. This could be due to essential oils present in the hexane extracts as shown previously in the family of Burseraceae [30].

**Figure 3:**
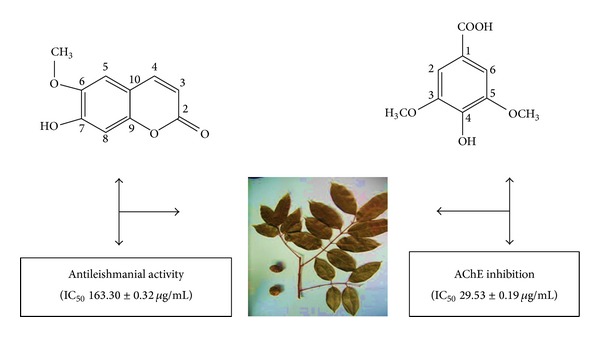
Graphical abstract of* Canarium patentinervium* Miq.

Scopoletin exhibited moderate antileishmanial activity (IC_50_ = 163.30 ± 0.32 *μ*g/mL) followed by lioxin, vomifoliol, scoparone, and (+)-catechin. Comparing the anti-AChE and antiparasitic activity, it can be deduced that scopoletin, scoparone, and (+)-catechin seemed to show correlation in both activities. Inhibitors of acetylcholinesterase (AChE) may decrease* Leishmania* survival by inhibiting choline formation from acetylcholine through hydrolysis. Again, the lactone groups present in the coumarins, namely, scopoletin and scoparone, are also present in the structures of* Annonaceous* acetogenins that showed leishmanicidal activity [10]. This might explain why scopoletin and scoparone showed correlation in both the assays. In fact, the AChE inhibitory activity of a previous study on scoparone indicated a possible mechanism of action by disrupting leishmania cell membranes [8]. There has been a hypothesis on the mechanism of action of coumarins acting on the same pathway as above compounds, resulting in net negative effects on choline uptake by the parasite [8]. Nevertheless, the antileishmanial activity of (+)-catechin may be related to its ability to chelate iron (Fe), depriving this essential nutrient from the intracellular forms [31]. However, lioxin and vomifoliol had lower anti-AChE activity but showed higher antileishmanial activity. Vomifoliol and lioxin both may be exhibiting their antileishmanial activity by mechanisms other than choline inhibition such as interference with the purine transporter as* Leishmania* species are unable to synthesize purines* de novo* and thus salvage these from their host. A graphical abstract of this study is shown in Figure 3.

## 4. Conclusion

This study is an important endeavour for the discovery of potent biologically active molecules for the treatment of neurodegenerative and parasitic diseases. Since only 12% of the 75 species of* Canarium* have been studied for their pharmacological activities, this study promises an unopened crypt of various secondary metabolites as lead compounds and various biological effects that needs to be uncovered and investigated. As modern cultures and scientific advances spread around the world, the depth of the knowledge store of traditional use still remains crucial. The full significance of the indigenous knowledge forfeited may not be realised. It is thus important that the knowledge be documented and the traditional use be given some credence through modern scientific studies.* Canarium patentinervium* Miq. is such an example.

## Figures and Tables

**Table 1:** Antiacetylcholinesterase values for crude extracts and isolated compounds from *Canarium patentinervium* Miq.

Samples	IC_50_ (*μ*g/mL)
Hexane extract of leaves	842.00 ± 0.25
Chloroform extract of leaves	1789.00 ± 0.24
Ethanol extract of leaves	201.34 ± 0.15
Hexane extract of barks	570.00 ± 0.08
Chloroform extract of barks	88.59 ± 0.14
Ethanol extract of barks	186.00 ± 0.15
Scopoletin	51.00 ± 0.02
Scoparone	86.58 ± 0.05
(+)-Catechin	>100
Vomifoliol	96.64 ± 0.09
Lioxin	>100
Syringic acid	29.53 ± 0.19
Galantamine	0.77 ± 0.09

Data were obtained from three independent experiments, each performed in triplicates (*n* = 9) and represented as mean ± SD. Values with the same capital letter are not significantly different (*P* < 0.01) according to Tukey multiple comparison test.

**Table 2:** Antileishmanial activity of crude extracts and compounds isolated from *Canarium patentinervium *Miq. against *Leishmania donovani *promastigotes.

Samples	IC_50_ (*μ*g/mL)
Hexane extract of leaves	257.40 ± 0.30
Chloroform extract of leaves	457.70 ± 0.25
Ethanol extract of leaves	>500
Hexane extract of barks	284.20 ± 0.40
Chloroform extract of barks	359.90 ± 0.20
Ethanol extract of barks	>500
Scopoletin	163.30 ± 0.32
Scoparone	329.90 ± 0.32
(+)-Catechin	478.93 ± 0.28
Vomifoliol	302.80 ± 0.33
Lioxin	211.48 ± 0.32
Syringic acid	nt
Amphotericin B	0.37 ± 0.10

Data were obtained from three independent experiments, each performed in triplicates (*n* = 9) and represented as mean ± SD. Values with the same capital letter are not significantly different (*P* < 0.01) according to Tukey multiple comparison test, nt: not tested.
